# Integrative bioinformatics analysis reveals STAT2 as a novel biomarker of inflammation-related cardiac dysfunction in atrial fibrillation

**DOI:** 10.1515/med-2023-0834

**Published:** 2023-11-09

**Authors:** Cairong Li, Guanhua Li, Sijia Tu, Xinghua Bai, Hong Yuan

**Affiliations:** Department of Cardiology, First People’s Hospital of Linping District, Hangzhou 311199, P.R. China; Department of Cardiology, First People’s Hospital of Linping District, 369 Yingbin Rd, Hangzhou 311199, P.R. China

**Keywords:** atrial fibrillation, COVID-19, WGCNA, scRNA-seq, STAT2

## Abstract

Atrial fibrillation (AF) is a common critical cause of stroke and cardiac dysfunction worldwide with lifetime risks. Viral infection and inflammatory response with myocardial involvement may lead to an increase in AF-related mortality. To dissect the potential sequelae of viral infection in AF patients, especially the coronavirus disease 2019 (COVID-19), based on AF and COVID-19 databases from Gene Expression Omnibus, weighted gene co-expression network analysis was used to identify key genes in heart tissues and peripheral blood mononuclear cells. Here, *HSCT*, *PSMB9*, *STAT2*, and *TNFSF13B* were identified as common risk genes of AF and COVID-19 patients. Correlation analysis of these genes with AF and COVID-19 showed a positive disease relevance. silencing of *STAT2* by small interfering RNA significantly rescued SARS-CoV-2 XBB1.5 pseudovirus-induced cardiac cell contraction dysfunction *in vitro*. In conclusion, we identified *STAT2* may be a novel biomarker of inflammation-related cardiac dysfunction in AF.

## Graphical abstract

**Figure:**
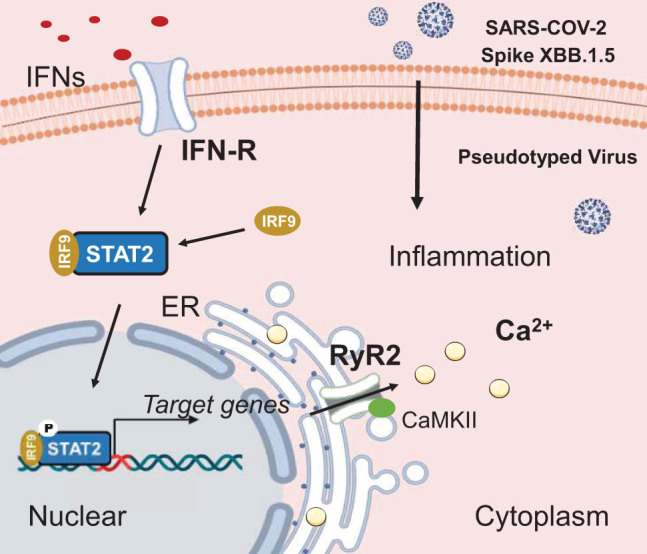

## 1 Introduction

Atrial fibrillation (AF) is one of the major contributors to the global cardiovascular disease burden with substantial morbidity and mortality. AF significantly increased the risk of embolic stroke and cardiac dysfunction [1,2]. Specifically, in the first 30 days after AF diagnosis, the risk of myocardial infarction (MI) in patients with AF is especially high [3,4,5]. Since its etiology and systemic changes are complex, the underlying mechanisms of the co-occurrence of AF and MI remain largely unclear. Several classic mechanisms have been reported in AF; most of them are cardiac-related, including the accumulation of underlying shared risk factors such as increased myocardial oxygen demand and coronary artery embolism [6,7]. However, viral infection and inflammatory response with myocardial involvement also play a pivotal role in AF-related mortality [8]. Thus, it is crucial to delve deeper into existing mechanisms and explore beyond them to comprehensively understand the initiation and progression of AF.

Studies have suggested that AF may be caused by several major signaling pathways. Inhibition of renin–angiotensin system can reduce NF-kB-mediated atrial fibrosis and cardiac dysfunction in AF progression [9,10]. Several studies have suggested that AF can exacerbate coronavirus disease 2019 (COVID-19) symptoms as well [11]. Some reports indicated that COVID-19 infection can also cause AF in some patients or exacerbate AF symptoms [6,12]. Nonetheless, molecular targets and pathogenesis mechanisms of AF’s interaction with COVID-19 remain unknown.

With the development of bioinformatics analyses based on high-throughput sequencing in the past decade, the identification of differentially expressed genes (DEGs) has improved our understanding of AF’s molecular mechanisms [13,14]. Bioinformatics analysis results are heavily influenced by the algorithm diversity. The weighted gene co-expression network analysis (WGCNA) identifies gene sets (gene modules) with similar expression patterns and identifies disease-related hub genes without DEG analysis [15]. In our current study, we integrated sequencing data of four AF tissue samples and one COVID-19 peripheral blood mononuclear cell (PBMC) sample from Gene Expression Omnibus (GEO) databases, identified functional gene modules, and highlighted the importance of prevention in AF.

## 2 Materials and methods

### 2.1 Data collection and preprocessing from the GEO database

Data were downloaded from the GEO database. The selected dataset details are shown in Table S1. According to the annotation information in the platform, probes were converted to gene symbols. We used the remove batch effect function of the limma package in the R software [16]. Principal component analysis (PCA) plots were drawn to depict the samples before and after batch effects. There are 14 AF tissue samples and 12 control tissue samples in the GSE79768 dataset, 12 control tissue samples in the GSE29819 dataset, 5 AF tissue samples in the GSE14975 dataset, and 32 AF tissue samples and 6 control tissue samples in the GSE41177 dataset. Moreover, there are 29 COVID-19 PBMC samples and 18 healthy PBMC samples in the GSE177477 dataset. In addition, there are 3 KD PBMC samples and 3 control PBMC samples in the GSE168732 dataset. In our study, the datasets from the GSE79768, GSE29819, GSE14975, and GSE41177 were used to screen DEGs in AF samples by building co-expression networks. The dataset GSE177477 was used to screen COVID-19 DEGs. The dataset GSE168732 was used to validate the function of hub genes in Kawasaki disease. The data procurement and application conform to the GEO databases’ principles and guidelines. For more information, refer to the supplement materials and methods.

### 2.2 DEGs’ identification and functional enrichment analysis

The DEGs between AF and normal samples, as well as COVID-19 and control samples, were identified using the R packages limma and edgeR [17,18]. Genes with an adjusted *P* < 0.05 and |log_2_(FC)| > 1 were selected as DEGs in heart tissues and PBMC samples, respectively. We acquired the shared DEGs using Venny.2, version 2.1.0, an online tool for VENN analysis. Gene Ontology (GO) and Kyoto Encyclopedia of Genes and Genomics (KEGG) analyses were conducted with Clusterprofiler and org.Hs.eg.db [19]. Terms with a *P* < 0.05 were considered statistically significant. By analyzing DEGs and WGCNA hub genes, we identified COVID-19 and AF shared pathways via Metascape (http://metascape.org/). For more information, refer to the supplement materials and methods.

### 2.3 WGCNA

The WGCNA package in R software was used to cluster the common modules on the AF and COVID-19 datasets [15]. A cut-off height of 0.25 was defined for merging modules that may be similar. The group phenotypes (AF/COVID-19) were inputted into the co-expression network. Genes with high hub modularity were considered hub genes in the modular-trait correlation analysis. A heatmap was then used to visualize the relationship between gene modules and clinical traits. After that, modules associated with phenotypes with co-expression patterns and significance were identified (darked module in AF, red module in COVID-19).

### 2.4 Analysis of single-cell sequencing dataset

We used single-cell transcriptome data of GSE168732 to validate the function of hub genes. Genes with fewer than three cells detected were excluded and genes with fewer than 200 detected genes were disregarded. Genes detected in each cell ranged from 0 to 8,000. A total of 34,412 cells were included in the study. In the construction of the UMAP, the top 20 PCAs were used as the standard for PCA construction. Through graph-based clustering, we obtained the unsupervised cell clustering result based on the top 20 principal functions. Then, we used the Average Expression function to calculate the average expression value of cells contained in cell subpopulations or different groups, and then calculate the score of each cell subpopulation or group’s corresponding pathway based on the GSVA package [20]. Finally, we visualize it through a pheatmap.

### 2.5 Cell culture

HL-1 (# FH1101) and AC16 (# FH1280) cells were obtained from Fuheng Biotechnology Co., Ltd. (Shanghai, China). H9c2 (# GNR 5) cells were obtained from the Cell Bank of the Chinese Academy of Science (Shanghai, China). All cells were performed short tandem repeat DNA profiling and verified as mycoplasma free. HL-1, AC16, and H9c2 cells were cultured in Dulbecco’s modified eagle medium supplemented with 10% fetal bovine serum. All cell lines were cultured in the presence of 100 units/ml penicillin (Thermo Fisher Scientific, 15140122) and 100 units/ml streptomycin (Thermo Fisher Scientific, 15240062) and incubated at 37°C in a humidified 5% CO_2_ chamber.

### 2.6 SARS-CoV-2-Spike-pseudovirus entry assay

All pseudovirus used in this study was purchased from Genomeditech, China (SARS-COV-2 Spike XBB.1.5 pseudotyped Virus GFP-Luciferase). The detailed method was described previously [21]. To treat cardiac cell lines, transfected cells were inoculated with pseudovirus for 48 h before cells were washed and lysed. In brief, 1 × 10^6^ cells were inoculated into 6 cm dishes for 24 h, followed by 1 μl diluted pseudovirus treatment. *In vitro* experiments involving SARS-CoV-2 XBB1.5 pseudovirus were performed in the Biosafety Level 3 laboratory and strictly followed the approved standard operation procedures. For more information and the raw data of pseudovirus, refer to the supplement materials and methods.

### 2.7 Total protein extraction and Western blotting

Cells were lysed in RIPA buffer with phosphatase inhibitors and protease inhibitors (Halt Protease and Phosphatase Inhibitor Cocktail; Thermo Fisher Scientific, 78440). Proteins were electrophoresed and transferred to PVDF membranes. After blocking with 5% non-fat milk for 1 h, a primary antibody was incubated with PVDF membranes overnight at 4°C. The secondary antibody was incubated for 45 min. Chemiluminescence kit (EpiZyme, China) was used for the protein signal visualizing. A list of all primary antibodies used in this study, as well as dilutions and vendor information, can be found in Table S2.

### 2.8 Statistics

A two-tailed independent Student’s test was used to assess statistical significance. The significance levels were *P* < 0.05 and *P* < 0.001; N.S., not significant.


**Ethics:** The Ethics Committee of First People’s Hospital of Linping District approved this study. Since all the data in the current study was available online, and no individual patient was involved, it could be confirmed we have obtained all the written informed consent.

## 3 Results

### 3.1 Identification of DEGs in heart tissue from AF datasets and PBMC from COVID-19 datasets

**Figure 1:**
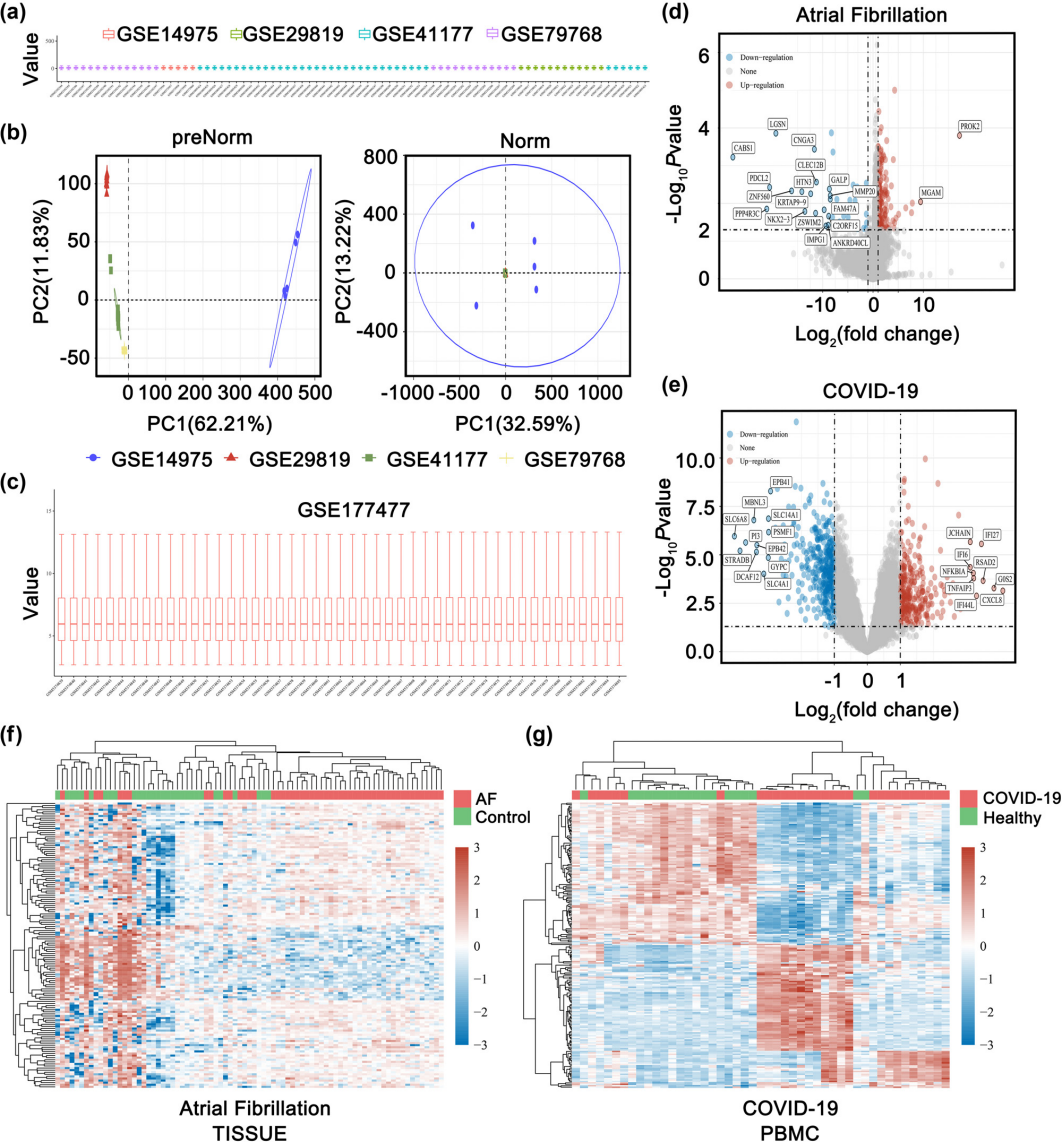
Identification of DEGs in AF tissue and COVID-19 PBMC. (a) The boxplot of the normalized AF data. Different colors represent different datasets. Rows represent samples, and columns represent the gene expression values in the samples. (b) PCA of the AF GSM datasets. The samples were visualized by scatter plots of gene expression profiles without (left) or with (right) batch effect removal. As shown in the schematic diagram shows the intersection of three datasets, which can be used in subsequent analysis. (c) The boxplot of the normalized COVID-19 data. (d) Volcano plots of the AF DEGs. Red dots indicate upregulated genes; blue dots indicate downregulated genes. (e) Volcano plots of the COVID-19 DEGs. Red dots indicate upregulated genes; blue dots indicate downregulated genes. (f) Heatmap showing AF DEGs in different samples. (g) Heatmap showing COVID-19 DEGs in different samples.

GEO datasets of AF patients and COVID-19 patients were used to extract transcriptome data. In this study, four tissue datasets (GSE79768, GSE29819, GSE14975, GSE41177) were included and analyzed to explore the critical genes in the heart tissue of AF patients. Since COVID-19 patients have a high incidence of AF, we used one PBMC dataset (GSE177477) with 29 COVID-19 patients and 18 healthy controls to screen for DEGs. Then, we used the R package ComBat to remove the batch effect. Boxplots and PCA showed the normalized GEO samples (GSMs) from different GEO datasets (Figure 1a–c). Different colors represent different datasets. The DEseq2 package was used to analyze the data for differential expression (log_2_FC > 2, adjusted *P* < 0.05). Preliminarily, we identified 191 DEGs in the AF datasets (Figure 1d) and 668 DEGs in the COVID-19 dataset compared to control samples (Figure 1e). Heatmaps showed the ranking of DEGs in AF (Figure 1f) and COVID-19 datasets (Figure 1g), respectively. Compared with controls, heart tissues from AF patients and PBMC samples from COVID-19 patients contained substantial DEGs.

### 3.2 Functional enrichment analysis of DEGs

**Figure 2:**
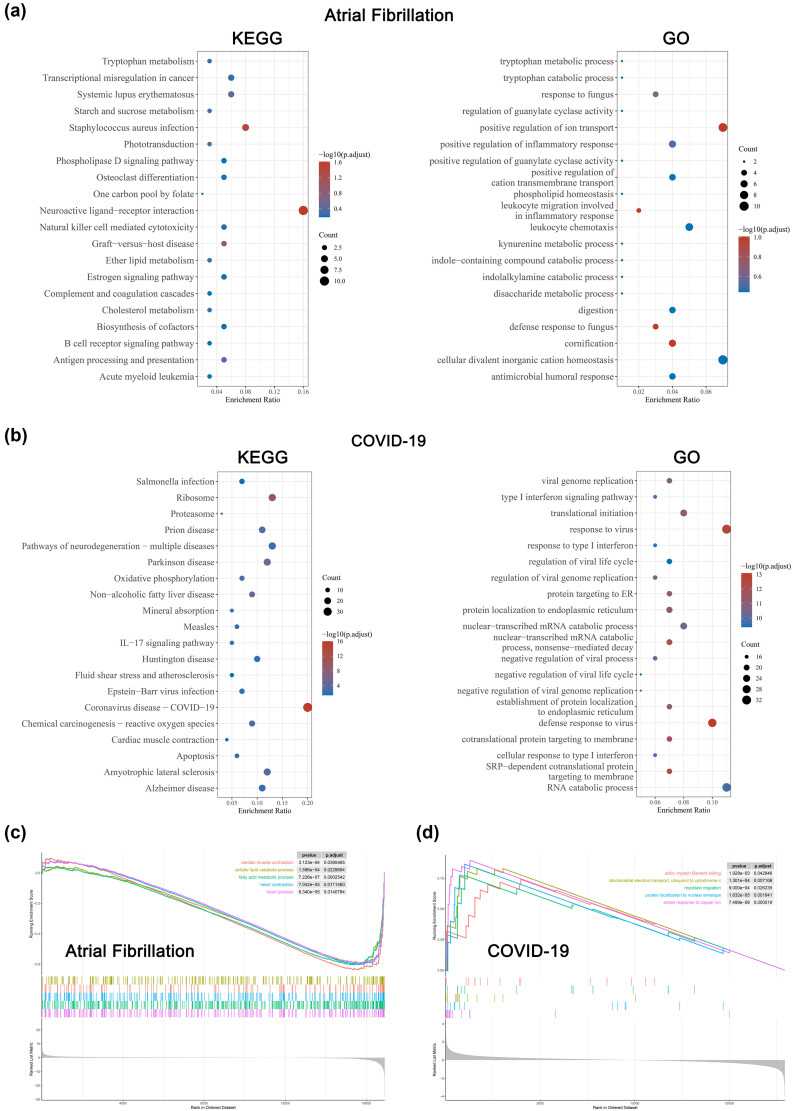
Functional enrichment analysis of DEGs. (a) The top 20 enriched KEGG (left) and GO (right) pathways among AF up-regulated DEGs. The gene ratio was represented on the horizontal axis. The vertical axis indicated the KEGG and GO signaling pathway terms, and the blue-to-red gradually changing color indicated the change of significance from low to high. (b) The top 20 enriched KEGG (left) and GO (right) pathways among COVID-19 upregulated DEGs. The gene ratio was represented on the horizontal axis. The vertical axis indicated the KEGG and GO signaling pathway terms, and the blue-to-red gradually changing color indicated the change of significance from low to high. (c) GSEA enrichment plot for the top 5 pathways in AF. Genes were ranked by Pearson correlation with AF in descending order. (d) GSEA enrichment plot for the top 5 pathways in COVID-19. Genes were ranked by Pearson correlation with COVID-19 in descending order.

To interpret the general biological properties involved in AF and COVID-19, we separately analyzed the upregulated DEGs of the AF group and the COVID-19 group via KEGG/GO analysis. The top KEGG/GO terms associated with AF (Figure 2a) and COVID-19 (Figure 2b) are shown presented. AF DEGs were enriched in positive regulation of inflammatory response, positive regulation of ion transport, tryptophan metabolism, and cholesterol metabolism, indicating the possible effect of inflammation status and energy metabolism in AF occurrence. COVID-19 DEGs were mainly involved in the defense response to viruses, such as coronavirus disease-COVID-19 and type I interferon signaling pathway. Besides, several intriguing signaling pathways were enriched, including fluid shear stress, atherosclerosis, and cardiac muscle contraction. Moreover, GSEA was performed using the top enriched DEGs of AF (Figure 2c) and COVID-19 (Figure 2d). Several signaling pathway terms upregulated in COVID-19 patients may be involved in AF development. Thus, it logically flows that if these traits could exacerbate AF symptoms, some of them may represent suitable targets for AF therapeutic intervention.

### 3.3 Identification and functional analysis of pivotal gene modules through WGCNA

To systematically clarify vital gene modules and potential mechanisms among heart tissue of AF and peripheral blood of COVID-19, after batch normalization, WGCNA was performed. A hierarchical clustering method was used to create multiple randomly color-coded modules for the cluster dendrogram (Figure 4a and b). The heatmaps are shown in Figure 4c (AF or control) and Figure 4d (COVID-19 or healthy). The darked module (corresponding correlation, CC = 0.41, *P* = 0.0002) showed the highest positive correlation with the AF trait in heart tissue. The red module (CC = 0.44, *P* = 0.002) showed a high positive correlation with COVID-19 traits in PBMC samples. Hub genes of the AF darked module including *HCST*, *BAX*, *GIMAP2*, *LCK*, *PSMB9*, and other 209 genes. Hub genes of the COVID-19 red module include *APOL6*, *STAT2*, *TNFSF13B*, *IFI6*, *MT1L*, and other 128 genes.

**Figure 3:**
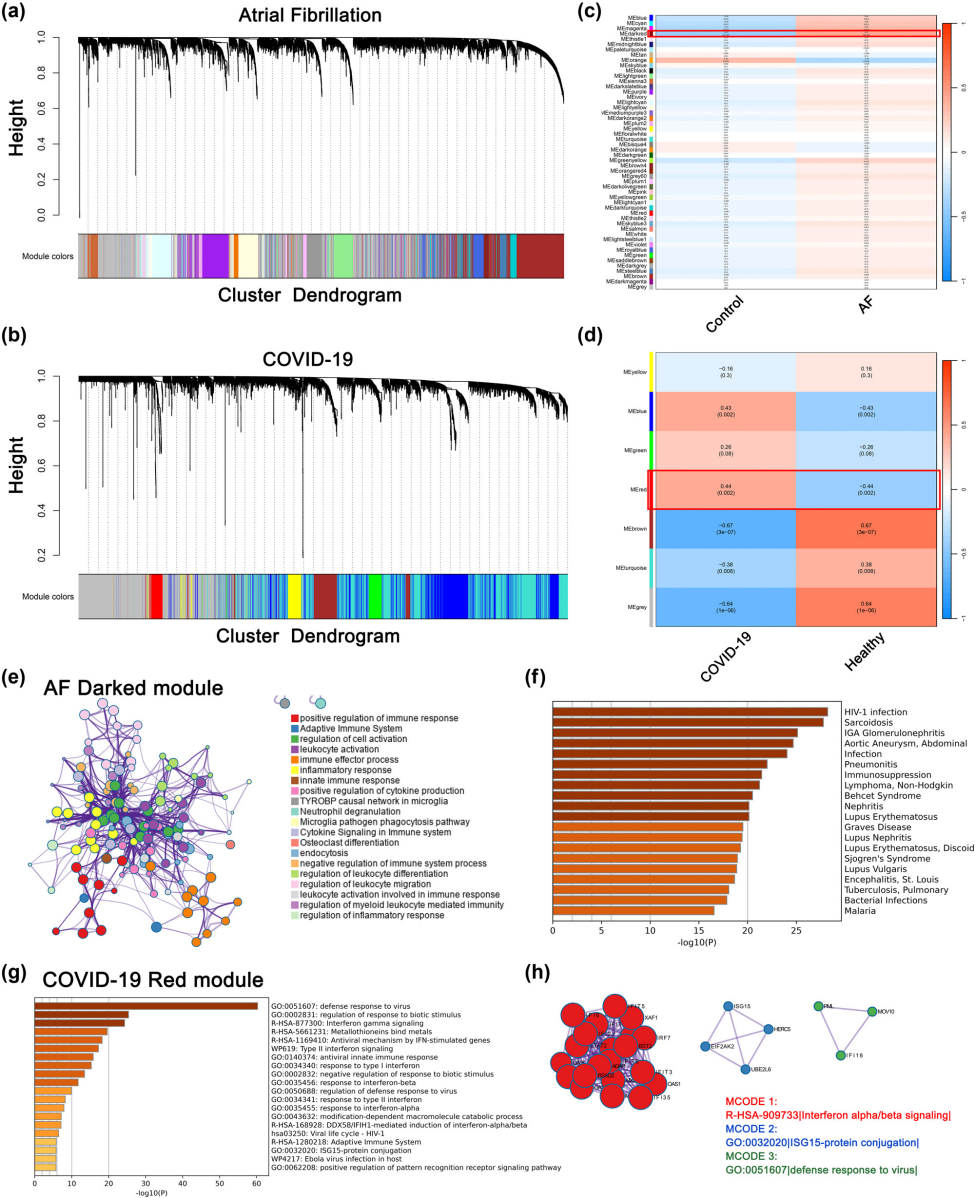
WGCNA revealing gene co-expression networks in AF and COVID-19 samples. (a) WGCNA analysis of AF samples. The dendrogram represented the clusters of DEGs based on different metrics. Each branch represented one gene, and each color below branches represented one co-expression module. (b) WGCNA analysis of COVID-19 samples. (c) The heatmap showed the correlation between gene modules and AF. The correlation coefficient in each cube represented the correlation between gene modules and traits, which decreased from red to blue. (d) The heatmap showed the correlation between gene modules and COVID-19. (e) The functional enrichment analysis of hub genes from the darked module. The horizontal axis represented *P*-value of GO terms on Metascape by default parameter. (f) Enriched DisGeNET terms among TISSUE hub genes. The horizontal axis represented *P*-value of GO terms on Metascape. (g) The functional enrichment analysis of hub genes from the red module. (h) Top MCODE terms of COVID-19 hub genes. PPI among COVID-19 hub genes from the red module formed a network. The Molecular Complex Detection algorithm (MCODE) was used to identify the connected network components.

The top GO terms of AF hub genes include positive regulation of immune response, adaptive Immune system, inflammatory response, and other immune-related pathways, suggesting the significant effect of the immune system in AF occurrence (Figure 3e). A DisNET analysis (Figure 3f) revealed the involvement of immune and inflammatory responses (HIV-1 infection, pneumonitis, and Immunosuppression). As for the hub genes of COVID-19, the top GO terms include defense response to virus, antiviral innate immune response, and regulation of defense response to virus (Figure 3g). Among them, The MCODE analysis enriched in interferon-alpha/beta signaling, ISG15-protein conjugation, and defense response to virus (Figure 3h). Taking together, the hub genes of AF may actively affect the immune system activation and inflammatory response, while the hub genes of COVID-19 are closely correlated with the defense response to virus.

### 3.4 Identification of common hub genes to assess the relationship between AF and COVID-19-associated inflammatory response

**Figure 4:**
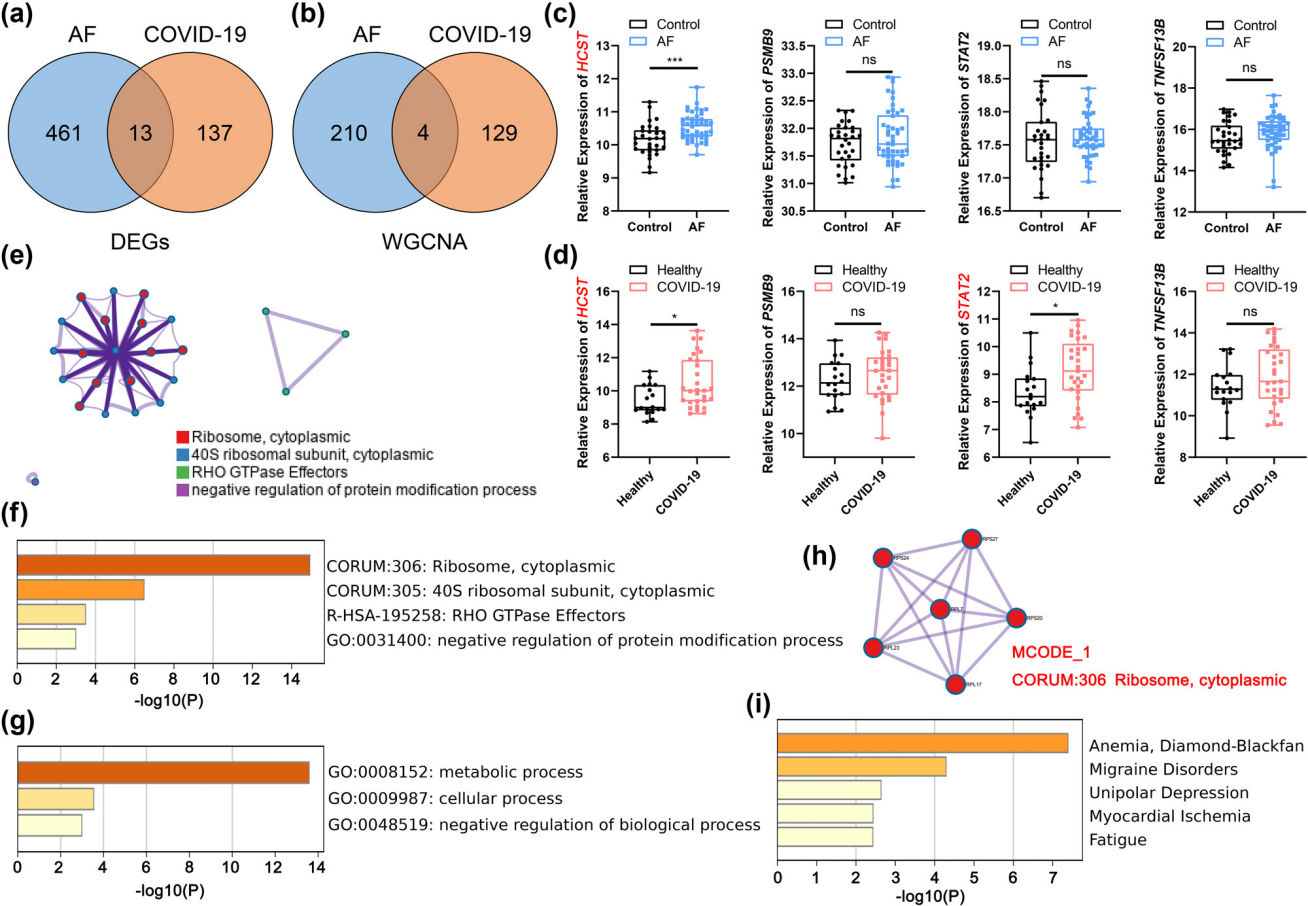
Common hub gene selection and enrichment analysis. (a) The common DEGs shared between AF and COVID-19 were visualized in a Venn diagram. (b) The common hub genes shared between AF and COVID-19 were visualized in a Venn diagram. (c) Estimation of hub gene expression in AF samples. (d) Estimation of hub gene expression in COVID-19 samples. *P < 0.05, ***P < 0.001, ns, *P* > 0.05. (e) The functional enrichment analysis of common DEGs shared between AF and COVID-19. (f) Network of representative GO terms among common DEGs. (g) GO pathway interaction network with each term. (h) Top MCODE terms of common DEGs. The Molecular Complex Detection algorithm (MCODE) was used to identify the connected network components. (i) Enriched DisGeNET terms of common DEGs.

To guide clinical decisions and stratify the condition of AF patients, the severity of SARS-CoV-2-driven AF must be accurately assessed. Specifically, 13 common genes in DEGs (Figure 4a) and 4 common genes in WGCNA (Figure 4b) were screened out and identified as key genes. As for the four common genes from WGCNA modules (*HCST*, *PSMB9*, *STAT2*, *TNFSF13B*), the *HCST* showed significant differences between the control/AF group (Figure 4c) and the healthy/COVID-19 group (Figure 4d), while the *STAT2* showed significant differences between the healthy and COVID-19 groups. We further performed gene enrichment analysis of thirteen common genes from DEGs by Metascape. The top Metascape terms were Ribosome, cytoplasmic, 40S ribosomal subunit, cytoplasmic, RHO GTPase Effectors, and negative regulation of protein modification process, showed by MCODE analysis (Figure 4e) and *P*-value (Figure 4f). In terms of GO analysis, negative regulation of biological processes, cellular processes, and metabolic processes were the top terms (Figure 4g). Furthermore, the MCODE analysis enriched Ribosome and cytoplasmic (Figure 4h). DisNET analysis revealed that DEG common genes were closely related to Myocardial Ischemia, which confirmed the corporate impact on cardiac tissue by AF progression and COVID-19 infection (Figure 4i).

### 3.5 Single-cell analysis re-validated the selected hub genes interacting with the inflammation-related cardiac dysfunction

As outlined in Section 1, prior research has identified shared underlying mechanisms between AF and COVID-19, specifically involving inflammation, immune response, and metabolic dysregulation. These findings suggest the involvement of inflammatory processes in the progression of AF. The primary objective of our study was to investigate the etiology and pathogenesis of cardiac dysfunction related to inflammation in individuals with AF. COVID-19, being a widespread infectious disease, has resulted in numerous fatalities globally. Its impact on infected individuals extends beyond respiratory complications, giving rise to various cardiovascular diseases in patients, especially AF. Furthermore, it is widely acknowledged that AF can be attributed to coronary artery lesions resulting from percutaneous coronary intervention (PCI) or inflammation response, as evidenced by studies [22,23]. However, most current researches primarily concentrate on the direct clinical risk assessment of coronary artery disease and AF, with no investigations reporting the potential molecular mechanisms underlying the involvement of coronary artery disease in the development and advancement of AF. Hence, our study aims to investigate the association and potential linkage between coronary artery disease and AF in Kawasaki disease (KD), a febrile systemic illness that can result in coronary artery disease and childhood vasculitis. This condition primarily affects children under the age of 5 and has emerged as the prevailing cause of acquired heart disease in children across numerous developed nations [24]. The age at which individuals are predisposed to KD aligns with the age group that is susceptible to COVID-19. Utilizing the single-cell sequencing data of KD, this study aims to validate the hub genes associated with inflammatory-related cardiac dysfunction, which were initially identified in the context of AF and COVID-19. The findings of this research have the potential to contribute significantly to the prevention of inflammatory-related cardiac dysfunction in the susceptible population of COVID-19, particularly among children aged below 5 years. Consequently, to gain further insights into the roles played by the common hub genes *HCST*, *PSMB9*, *STAT2*, and *TNFSF13B* in inflammation-related cardiac dysfunction, an analysis was conducted on a cohort comprising three control subjects and three patients diagnosed with KD.

**Figure 5:**
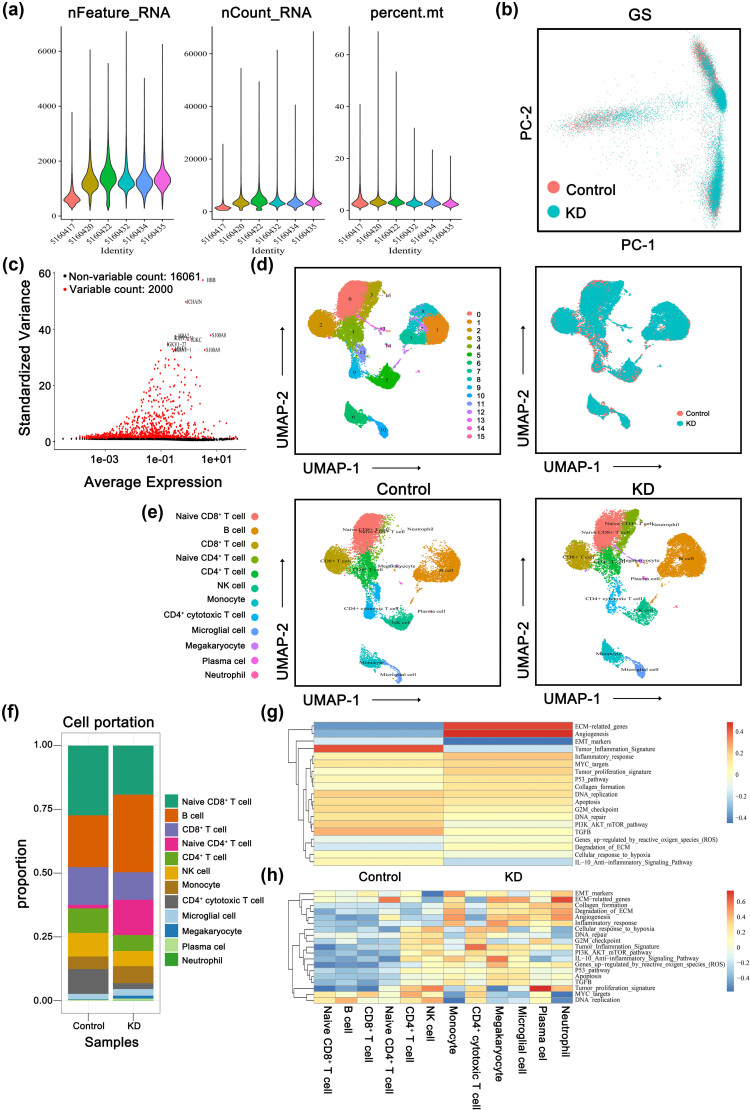
Analysis of single-cell RNA sequencing data from 34,412 cells of 3 control patients and 3 KD patients. (a) The gene counts per cell (nFeature_RNA), number of unique molecular identifiers (UMIs) per cell (nCount_RNA), and percentage of mitochondrial genes per cell (percent.mt) of the single-cell RNA-seq data. (b) PCA of the scRNA-seq data. The samples were visualized by scatter plots of gene expression profiles with batch effect removal. (c) The variance plot showed 16,061 genes in all cells, red dots represent the top 2000 highly variable genes. (d) Cells were divided into 15 separate clusters by UMAP. (e) Cells were clustered into 12 types via UMAP dimensionality reduction algorithm, each color represented the annotated phenotype of each cluster. (f) The distribution of cell proportions in different groups, with the horizontal axis representing different groups and the vertical axis representing the proportion of each type of cell. (g) Heatmap showing different pathways enriched in control and KD by GSVA. Each column represents different groups or subpopulations of cells, and each row represents a pathway. The redder the color, the higher the score, and the bluer the color, the lower the score. (h) Heatmap showing different pathways enriched in cell subpopulations by GSVA.

To examine the robustness of these genes and whether their expression influenced KD progression through inflammation response. Gene expression profiles from six samples were obtained from the GSE168732 dataset. As shown in Figure 5a and b, after rationalizing the sequencing depth, the number of detected genes, and the normalization of selected data, we selected 2,000 highly variable genes for further analysis. (Figure 5c). The “RunPCA” function was used to reduce the dimensionality, and 15 clusters were identified at a resolution of 0.5 (Figure 5d). Subsequently, the “SingleR” function was used for cell annotation, and 12 cell subgroups, such as B cells, NK cells, monocytes, and CD8^+^ T cells were annotated and visualized according to their marker genes (Figure 5e). The distribution of different cell types in the two groups was performed in Figure 5f, and we noticed that the B cells, naive CD4^+^ T cells, monocytes, megakaryocytes, plasma cells, and neutrophils took a larger proportion in KD compared with the control. In addition, DEGs between the control and KD groups were highly enriched for inflammatory pathways, such as inflammatory response, tumor inflammation signature, and IL-10 anti-inflammatory signaling pathway (Figure 5g). Of interest, we found that in the megakaryocyte cluster, inflammatory-related pathways such as inflammatory response and IL-10 anti-inflammatory signaling pathway were both significantly up-regulated (Figure 5h). In brief, immune activation provides important evidence for KD pathogenesis.

**Figure 6:**
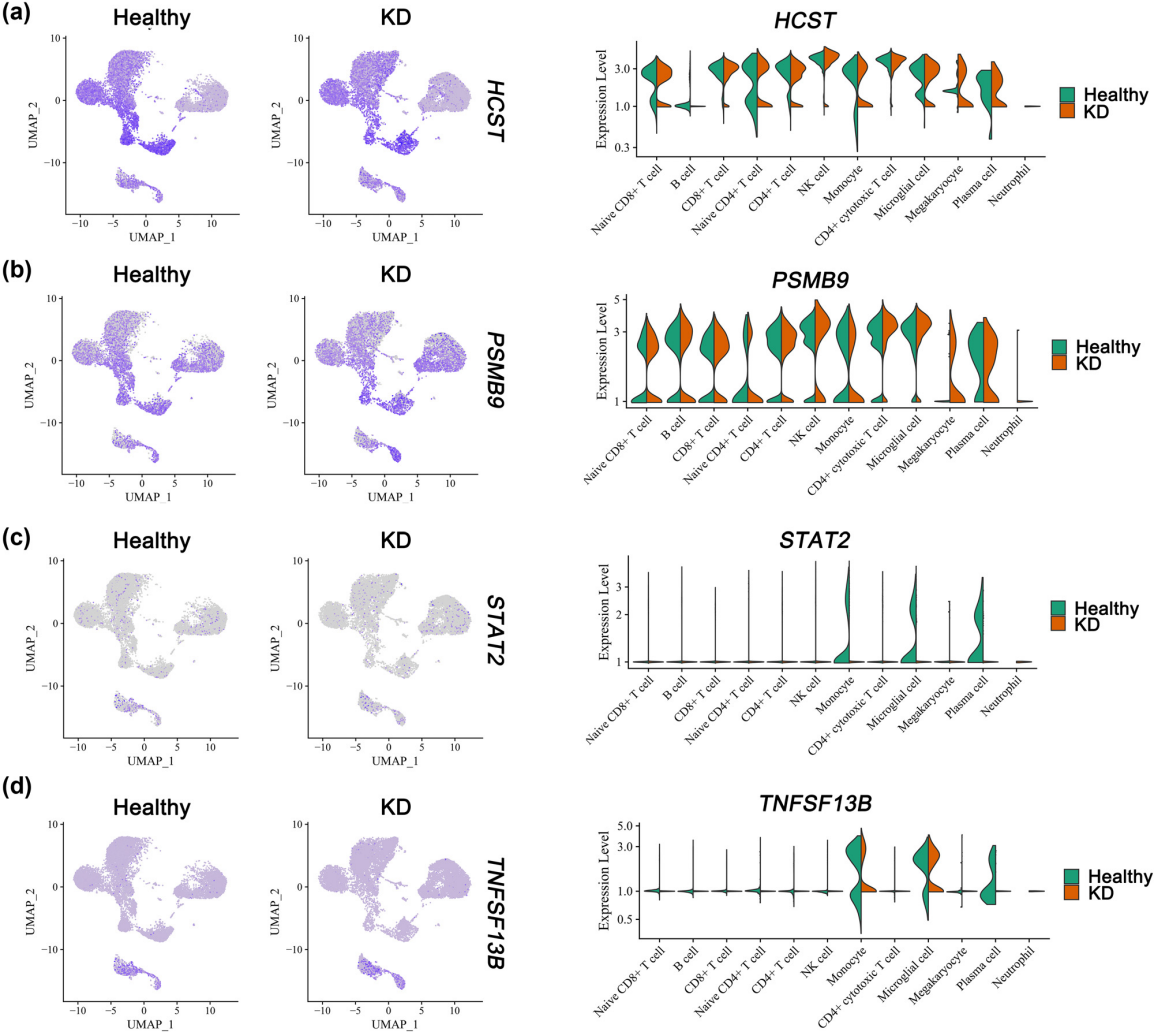
Cell-specific expression of selected hub genes in Kawasaki disease based on single-cell RNA sequencing data. (a) Feature and violin plots showing the distribution of HCST in phenotype (left) and various cell subpopulations (right). (b) Feature and violin plots showing the distribution of PSMB9 in phenotype (left) and various cell subpopulations (right). (c) Feature and violin plots showing the distribution of STAT2 in phenotype (left) and various cell subpopulations (right). (d) Feature and violin plots showing the distribution of TNFSF13B in phenotype (left) and various cell subpopulations (right).

Furthermore, the distribution and expression of selected common hub genes (*HCST*, *PSMB9*, *STAT2*, and *TNFSF13B*) in different cell types are shown in Figure 6a–d. We noticed the *STAT2* and *TNFSF13B* were both found to be correlated with the monocytes, microglial cells, and plasma cells of the healthy control group than the KD group compared with other immune cell types, while the *HCST* and *PSMB9* were highly expressed in all immune cell types except the neutrophil, indicating the involvement of our selected hub genes in inflammatory response. Owing to the critical role of *HCST* and *STAT2* in the inflammatory response, we further explored the potential mechanism regulated by these two hub genes after virus infection in myocardial cells.

### 3.6 *In vitro* validation of the inflammation-related cardiac dysfunction genes

**Figure 7:**
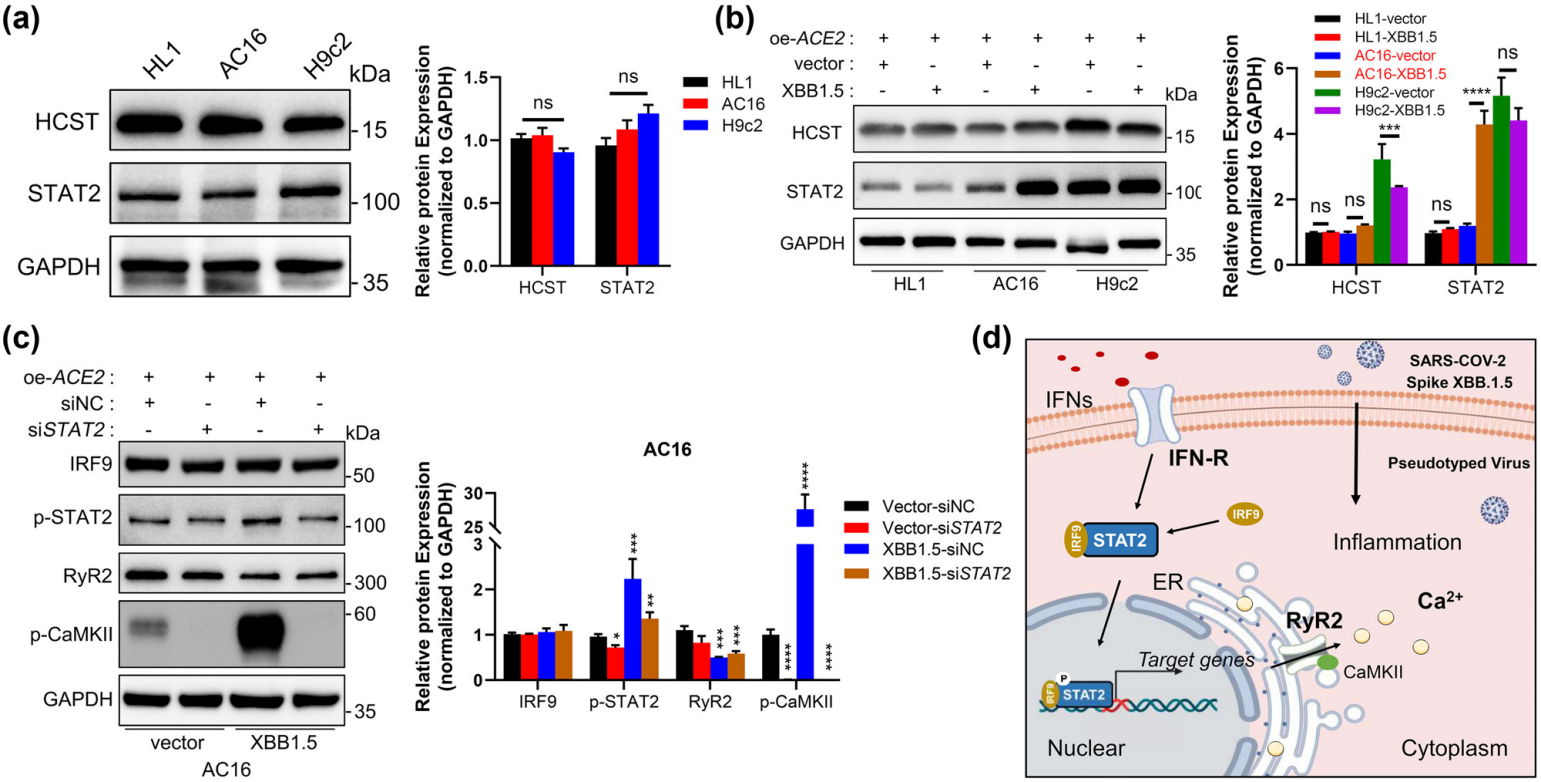
*In vitro* validation of the inflammation-related cardiac dysfunction genes. (a) Analysis of the HCST and STAT2 relative protein levels in indicated cardiac cell lines by Western blotting. (b) Western blotting analysis (left) and statistical analysis (right) of the indicated proteins in indicated cardiac cell lines treated with SARS-CoV-2 XBB1.5 pseudovirus for 48 h. (c) Western blotting analysis (left) and statistical analysis (right) of the cardiomyocyte contraction-related protein levels in AC16 cells with or without siRNA treatment. (d) Schematic diagram delineating the mechanism by which STAT2 exerts its pro-inflammation function in AF.

Since the current virus strain prevalent in China mainland is SARS-CoV-2 XBB1.5 [25], to mimic inflammation-related cardiac dysfunction during SARS-CoV-2 infection, we developed an XBB1.5 pseudovirus-induced cardiac cell model *in vitro*. We preferentially analyzed the basic expression levels of HCST and STAT2 in HL1 (mouse), AC16 (human), and H9c2 (rat) cardiac cell lines (Figure 7a). To further explore the influence of XBB1.5 pseudovirus treatment on cardiac cells, we overexpressed exogenous *ACE2* in these cells, which has been reported as the main receptor of SARS-CoV-2 virus. The results showed the expression changes in HCST and STAT2 in the cell lines mentioned above after 48 h of SARS-CoV-2 XBB1.5 pseudovirus treatment. The protein levels of HCST and STAT2 in cardiac cells were significantly increased after pseudovirus treatment. Among them, the level of STAT2 in AC16 cells was most affected by pseudovirus treatment, which indicated the COVID-19 vulnerability of human species (Figure 7b). Based on our functional analysis, inflammation-related cardiac dysfunction in AF was linked to heart cell contraction. Therefore, we hypothesized that the STAT2-associated mechanism may have a role in reducing cardiomyocyte contraction after SARS-CoV-2 infection. The results showed that RyR2 level was significantly reduced and p-CaMKII significantly increased after pseudovirus treatment, which related to cell contraction related to calcium ion pathways, indicating the dysfunction of contraction in cardiac cells. Interestingly, Figure 7c shows the changes in cell contraction biomarkers could be rescued by *STAT2* knocked down using small-interfering RNA (siRNA) [26]. However, the RyR2 levels were much lower in the XBB1.5 treatment group with or without *STAT2* knockdown, indicating that the XBB1.5 treatment itself could downregulate the RyR2 expression in cardiomyocytes without altering *STAT2*. Besides, comparing the RyR2 expression levels after XBB1.5 treatment in the siNC group with the si*STAT2* group, the knockdown of *STAT2* can partially rescue the decrease of RyR2 expression caused by XBB1.5 treatment, although there is no statistical difference. Collectively, our *in vitro* experiment results indicated that knockdown of *STAT2* can lead to downregulation of RyR2 expression in normal conditions, while knockdown of *STAT2* after XBB1.5 treatment can contrariwise rescue the downregulation of RyR2, highlighting the bidirectional role of STAT2 in the two different conditions.

More importantly, our results demonstrated that after *STAT2* knockdown, the expression of p-CaMKII was significantly inhibited, indicating that STAT2’s regulation on RyR2 was more likely mediated by calcium-calmodulin (CaM)-dependent activity. In our study, the increased expression level of p-CaMKII in XBB1.5 treatment implies a potential risk of AF induction. The knockdown of *STAT2* significantly inhibited the upregulation of p-CaMKII induced by XBB1.5 treatment, suggesting the potential value of STAT2 as a preventive measure for inflammatory-related cardiac dysfunction. Therefore, we look forward to further elucidating the value of STAT2-mediated calcium calmodulin-dependent activity in preventing the progression of AF in our future research. Collectively, these data highlighted the important role of STAT2 in cardiomyocyte contraction failure after SARS-CoV-2 infection, which might provide novel insights for the prevention of inflammation-related cardiac dysfunction in AF.

## 4 Discussion

Considering the dismal prognosis of AF patients after being infected with COVID-19 reported by multiple clinical studies, it is critical to understand the potential mechanism and identify novel therapeutic targets for this disease. It is known that the underlying inflammatory status usually leads to more severe clinical symptoms and higher mortality in AF, which is related to embolism and hemodynamic disturbances by arrhythmia [27,28]. Besides, pre-existing AF exacerbates the COVID-19 infection symptoms and increases mortality in patients, which indicates the strong link between COVID-19 and AF [29,30]. Our study examined the gene expression profiles of heart tissues from AF patients and PBMC from COVID-19 patients using multiple bioinformatics methods (Figure 7d).

In the present study, using multiple bioinformatics methods, we analyzed the gene expression profiles of heart tissues from AF patients and PBMCs from COVID-19 patients. In the primary screening, we identified 154 upregulated DEGs and 37 downregulated DEGs in AF datasets, and 293 upregulated DEGs and 375 downregulated DEGs in the COVID-19 dataset. Several signaling pathways were found to be significantly enriched within DEGs. Specifically, signal pathways such as inflammatory response and energy metabolism played critical roles in AF progression, and pathways related to defense response to viruses were involved in COVID-19 occurrence. Moreover, WGCNA is considered a better method to identify internal functional modules among key genes than DEG analysis [31]. However, few studies using WGCNA to combine both heart tissues and PBMC data have been performed. Next, based on the AF and COVID-19 data, we identified pivotal two gene modules. Genes in the two modules both play important roles in the occurrence of AF and COVID-19. Then, four genes were identified through a comprehensive analysis of WGCNA in AF and COVID-19 and validated with single-cell analysis and *in vitro* experiments. Finally, we found that *STAT2* was mainly expressed in AF and COVID-19 patients, respectively. *STAT2* knocked down could rescue the SARS-CoV-2 infection-related cardiac cell contraction dysfunction. These results may provide novel insights into the inflammation-related cardiac dysfunction in AF progression.

SARS-CoV-2, a virus causing COVID-19, has been linked to an inflammatory immune response and is associated with AF progression [3,32]. However, the pathogenesis of AF concomitance with SARS-CoV-2 infection remains unclear. We aimed to find the possible common pathophysiological pathways among AF and other inflammation-related diseases, especially COVID-19, including KEGG and GO terms that can identify the biological processes of shared genes and possible common molecular links among AF and inflammation-related diseases. Previous studies have reported potential pathways. In AF patients, calcium disturbances play an important pathological role in arrhythmias new onset [33,34]. Specifically, there is a link between calcium disorders and myocardial injury, and calcium is essential for viral structure formation, entry, gene expression, viral maturation, and viral release [35]. Therefore, when calcium consumption and calcium concentrations are disturbed in AF patients, the virus could more easily get into the heart tissue and further depletion of calcium storage in myocardial cells, which formed worsening feedback of cardiac dysfunction. Therefore, calcium disorders may be the critical reason for inflammation-related cardiac dysfunction in AF.

To date, several studies have reported key markers of COVID-19-related AF [36,37,38]. In our study, four critical genes (*HCST*, *PSMB9*, *STAT2*, and *TNFSF13B*) were identified by WGCNA. Several of these genes have been reported in molecular and pathology changes in COVID-19 and AF progress. HCST (also known as DAP10) is a membrane-associated signal adaptor harboring a costimulatory YINM motif which may activate the phosphatidylinositol 3-kinase-dependent signaling pathway [39]. HCST is expressed in NK cells and CD8 T cells and functions by interacting with NKG2D. HCST directly binds to the Receptor for Advanced Glycation End Products and modulates the S100A8/A9-triggered signaling pathway. Previous studies have identified the *HCST* as a key immune-related gene involved in m^6^A regulator-mediated RNA methylation modification patterns in AF, suggesting its potential value as an intervention target in the AF immune microenvironment under the COVID-19 burden [39,40].

Besides, PSMB9 (alias LMP2) is an interferon-gamma inducible factor and the gene is located within the MHC class II region in chromosome 6 [41]. PSMB9 degrades damaged or unneeded proteins by breaking peptide bonds and may limit the presentation and processing of antigens, making the *PSMB9* gene an attractive candidate for raising cancer risk. Moreover, a previous clinical trial has reported that oral proteasome inhibitor ixazomib could downregulate proteasome gene *PSMB9* in patients with indolent B-cell non-Hodgkin lymphoma and help to generate anti-S antibodies to SARS-CoV-2 vaccination, indicating the potential relation between PSMB9 expression and COVID-19 infection [42].

In addition, TNFSF13B is a tumor necrosis factor superfamily member encoded on the *TNFSF13B* gene. The cytokine is an effective B-cell activator that plays an important role in B-cell homeostasis, differentiation, and survival [43]. Studies on the comprehensive analysis of a ceRNA immunoregulatory network and tissue-infiltrating immune cells in AF have revealed the association between TNFSF13B and immune cells activated by Tregs or NK cells and provided new insights into the molecular mechanisms governing AF progression from the perspective of inflammatory response [43,44].

Moreover, there have been some studies that suggest an association between *STAT2* expression and AF/COVID progression and have illuminated their heart-specific roles in arrhythmias, but the exact mechanisms underlying inflammation-related cardiac dysfunction in AF are still elusive. Of note, STAT2 is a signal transducer and activator of transcription that is considered a hallmark of INF-I activation [45,46]. Other studies suggested that *STAT2* deficiency underlies inflammatory viral diseases and is related to multisystem inflammatory syndrome [47,48]. MacCann and his colleagues have determined that early host immune-related gene expression including type I interferon (IFN) signaling (*STAT2*, *IRF4*, *PML*, *BST2*, etc.) could predict clinical progression to COVID-19 infection [49]. Consistent with the conclusions of the research mentioned above, our results highlighted the inflammation response related to STAT2 in AF and COVID-19 data by KEGG/GO analysis. Furthermore, there is a positive correlation between STAT2 expression and monocytes, microglial cells, and plasma cells of the healthy control group than the KD group, which indicates the important role of STAT2 in the immune response through its transcriptional activity. Our results indicated that *STAT2* knocked down could rescue the decreased expression of cardiac contraction biomarkers induced by SARS-CoV-2 XBB1.5 pseudovirus treatment, which provided a theoretical basis for the use of STAT family inhibitors such as NSC74859 [50], Fludarabine [51], and Stattic [52] to alleviate inflammation-related cardiac dysfunction in patients with AF caused by viral infection via blocking the transcription and signal transduction functions of STAT2. Moreover, since pre-existing AF could aggravate the symptoms of COVID-19 infection, inspired by antibiotic pretreatment, we believe that on the premise of ensuring safety and drug compatibility, the use of STAT inhibitors in advance for AF patients in an infection-risk environment might provide potential clinical benefits for the risk of inflammatory cardiac dysfunction (AF, ventricular fibrillation, heart failure, etc.). However, this hypothesis requires more large-scale clinical trial studies for further exploration and validation. Thus, the study of exploration of the effect of STAT2 on inflammation-related cardiac dysfunction caused by COVID-19 infection in patients with AF should therefore be intensified.

Also, like all scientific researches, our study had some limitations. For example, we analyzed gene mRNA levels but not protein levels of the online datasets. For sample selection, the lack of control sample for normal cardiac tissue in each single AF cardiac sample is a major limitation in our study. Due to the preciousness of the cardiac tissue, we are currently unable to address this issue. In addition, we only conducted comparisons between COVID-19 patients and healthy control PBMCs and lacked data on COVID-19 patients before and after infection, which is also one of the limitations of current research. In addition, we only performed the *in vitro* experiments to validate the function of the hub gene in SARS-CoV-2-driven AF due to the consideration of laboratory safety since the COVID-19 virus is still an epidemic disease that poses a risk to humans. Hence, more work and further validation in multicenter, large-sample cohorts are urgently needed.

## 5 Conclusion

In this study, we explored the potential mechanisms of inflammation-related cardiac dysfunction in AF and identified calcium regulation pathways as possible mechanisms linking COVID-19 with AF. We performed WGCNA analysis and found that hub gene STAT2 may be a key regulatory molecule. We validated the effect of STAT2 on contractile function in myocardial cells after COVID-19 infection by single-cell analysis and *in vitro* experiments. Our work provides new insights into underlying mechanisms of inflammation-related cardiac dysfunction in AF.

## Supplementary Material

Supplementary material

## References

[1] Andrade J, Khairy P, Dobrev D, Nattel S. The clinical profile and pathophysiology of atrial fibrillation: relationships among clinical features, epidemiology, and mechanisms. Circ Res. 2014;114(9):1453–68. 10.1161/circresaha.114.303211. Epub 2014/04/26. PubMed PMID: 24763464. 24763464

[2] Heijman J, Algalarrondo V, Voigt N, Melka J, Wehrens XH, Dobrev D, et al. The value of basic research insights into atrial fibrillation mechanisms as a guide to therapeutic innovation: a critical analysis. Cardiovasc Res. 2016;109(4):467–79. 10.1093/cvr/cvv275. Epub 2015/12/26. PubMed PMID: 26705366; PubMed Central PMCID: PMCPMC4777910. PMC4777910 26705366

[3] Jering KS, Claggett BL, Pfeffer MA, Granger CB, Køber L, Lewis EF, et al. Prognostic importance of NT-proBNP (N-terminal Pro-B-type natriuretic peptide) following high-risk myocardial infarction in the PARADISE-MI trial. Circ Heart Fail. 2023;16(5):e010259. 10.1161/circheartfailure.122.010259. Epub 2023/05/01. PubMed PMID: 37125529. 37125529

[4] January CT, Wann LS, Alpert JS, Calkins H, Cigarroa JE, Cleveland JC, Jr, et al. 2014 AHA/ACC/HRS guideline for the management of patients with atrial fibrillation: a report of the American College of Cardiology/American Heart Association Task Force on practice guidelines and the Heart Rhythm Society. Circulation. 2014;130(23):e199–267. 10.1161/cir.0000000000000041. Epub 2014/04/01. PubMed PMID: 24682347; PubMed Central PMCID: PMCPMC4676081. PMC4676081 24682347

[5] Rudolph V, Andrié RP, Rudolph TK, Friedrichs K, Klinke A, Hirsch-Hoffmann B, et al. Myeloperoxidase acts as a profibrotic mediator of atrial fibrillation. Nat Med. 2010;16(4):470–4. 10.1038/nm.2124. Epub 2010/03/23. PubMed PMID: 20305660; PubMed Central PMCID: PMCPMC2880896. PMC2880896 20305660

[6] Stone E, Kiat H, McLachlan CS. Atrial fibrillation in COVID-19: A review of possible mechanisms. FASEB J. 2020;34(9):11347–54. 10.1096/fj.202001613. Epub 2020/10/21. PubMed PMID: 33078484. 33078484

[7] Kume O, Takahashi N, Wakisaka O, Nagano-Torigoe Y, Teshima Y, Nakagawa M, et al. Pioglitazone attenuates inflammatory atrial fibrosis and vulnerability to atrial fibrillation induced by pressure overload in rats. Heart Rhythm. 2011;8(2):278–85. 10.1016/j.hrthm.2010.10.029. Epub 2010/11/03. PubMed PMID: 21034856. 21034856

[8] Paris S, Inciardi RM, Lombardi CM, Tomasoni D, Ameri P, Carubelli V, et al. Implications of atrial fibrillation on the clinical course and outcomes of hospitalized COVID-19 patients: results of the Cardio-COVID-Italy multicentre study. Europace. 2021;23(10):1603–11. 10.1093/europace/euab146. Epub 2021/07/24. PubMed PMID: 34297833; PubMed Central PMCID: PMCPMC8344555. PMC8344555 34297833

[9] Marrouche NF, Wilber D, Hindricks G, Jais P, Akoum N, Marchlinski F, et al. Association of atrial tissue fibrosis identified by delayed enhancement MRI and atrial fibrillation catheter ablation: the DECAAF study. Jama. 2014;311(5):498–506. 10.1001/jama.2014.3. Epub 2014/02/06. PubMed PMID: 24496537. 24496537

[10] Gao G, Dudley SC, Jr. Redox regulation, NF-kappaB, and atrial fibrillation. Antioxid Redox Signal. 2009;11(9):2265–77. 10.1089/ars.2009.2595. Epub 2009/03/25. PubMed PMID: 19309257; PubMed Central PMCID: PMCPMC2819799. PMC2819799 19309257

[11] Shi S, Qin M, Shen B, Cai Y, Liu T, Yang F, et al. Association of cardiac injury with mortality in hospitalized patients With COVID-19 in Wuhan, China. JAMA Cardiol. 2020;5(7):802–10. 10.1001/jamacardio.2020.0950. Epub 2020/03/27. PubMed PMID: 32211816; PubMed Central PMCID: PMCPMC7097841. PMC7097841 32211816

[12] Abdulrahman A, Hussain T, Nawaz S, AlShaikh S, Almadani A, Bardooli F. Is atrial fibrillation a risk factor for worse outcomes in severe COVID-19 patients: A single center retrospective cohort. J Saudi Heart Assoc. 2021;33(2):160–8. 10.37616/2212-5043.1255. Epub 2021/07/22. PubMed PMID: 34285872; PubMed Central PMCID: PMCPMC8274695. PMC8274695 34285872

[13] Ou SM, Tsai MT, Chen HY, Li FA, Tseng WC, Lee KH, et al. Identification of galectin-3 as potential biomarkers for renal fibrosis by RNA-sequencing and clinicopathologic findings of kidney biopsy. Front Med (Lausanne). 2021;8:748225. 10.3389/fmed.2021.748225. Epub 2021/12/07. PubMed PMID: 34869439; PubMed Central PMCID: PMCPMC8633540. PMC8633540 34869439

[14] Lu Y, Zhao N, Du Y. Comprehensive bioinformatics analysis reveals common potential mechanisms, progression markers, and immune cells of coronary virus disease 2019 and atrial fibrillation. Front Cardiovasc Med. 2022;9:1027026. 10.3389/fcvm.2022.1027026. Epub 2022/11/11. PubMed PMID: 36352845; PubMed Central PMCID: PMCPMC9637541. PMC9637541 36352845

[15] Langfelder P, Horvath S. WGCNA: an R package for weighted correlation network analysis. BMC Bioinform. 2008;9:559. 10.1186/1471-2105-9-559. Epub 2008/12/31. PubMed PMID: 19114008; PubMed Central PMCID: PMCPMC2631488. PMC2631488 19114008

[16] Leek JT, Johnson WE, Parker HS, Jaffe AE, Storey JD. The sva package for removing batch effects and other unwanted variation in high-throughput experiments. Bioinformatics. 2012;28(6):882–3. 10.1093/bioinformatics/bts034. Epub 2012/01/20. PubMed PMID: 22257669; PubMed Central PMCID: PMCPMC3307112. PMC3307112 22257669

[17] Ritchie ME, Phipson B, Wu D, Hu Y, Law CW, Shi W, et al. Limma powers differential expression analyses for RNA-sequencing and microarray studies. Nucleic Acids Res. 2015;43(7):e47. 10.1093/nar/gkv007. Epub 2015/01/22. PubMed PMID: 25605792; PubMed Central PMCID: PMCPMC4402510. PMC4402510 25605792

[18] Robinson MD, McCarthy DJ, Smyth GK. edgeR: a Bioconductor package for differential expression analysis of digital gene expression data. Bioinformatics. 2010;26(1):139–40. 10.1093/bioinformatics/btp616PubMed PMID: 19910308; PubMed Central PMCID: PMCPMC2796818. Epub 2009/11/17. PMC2796818 19910308

[19] Yu G, Wang LG, Han Y, He QY. clusterProfiler: an R package for comparing biological themes among gene clusters. Omics. 2012;16(5):284–7. 10.1089/omi.2011.0118. Epub 2012/03/30. PubMed PMID: 22455463; PubMed Central PMCID: PMCPMC3339379. PMC3339379 22455463

[20] Hänzelmann S, Castelo R, Guinney J. GSVA: gene set variation analysis for microarray and RNA-seq data. BMC Bioinform. 2013;14:7. 10.1186/1471-2105-14-7. Epub 2013/01/18. PubMed PMID: 23323831; PubMed Central PMCID: PMCPMC3618321. PMC3618321 23323831

[21] Bi Z, Hong W, Que H, He C, Ren W, Yang J, et al. Inactivated SARS-CoV-2 induces acute respiratory distress syndrome in human ACE2-transgenic mice. Signal Transduct Target Ther. 2021;6(1):439. 10.1038/s41392-021-00851-6. Epub 2021/12/26. PubMed PMID: 34952899; PubMed Central PMCID: PMCPMC8705082. PMC8705082 34952899

[22] Alexandre J, Saloux E, Chequel M, Allouche S, Ollitrault P, Plane AF, et al. Preoperative plasma aldosterone and the risk of atrial fibrillation after coronary artery bypass surgery: a prospective cohort study. J Hypertens. 2016;34(12):2449–57. 10.1097/hjh.0000000000001105. Epub 2016/11/03. PubMed PMID: 27584972. 27584972

[23] Pokorney SD, Berchuck SI, Chiswell K, Sun JL, Thomas L, Jones WS, et al. Atrial branch coronary artery stenosis as a mechanism for atrial fibrillation. Heart Rhythm. 2022;19(8):1237–44. 10.1016/j.hrthm.2021.12.020. Epub 2021/12/28. PubMed PMID: 34958941. 34958941

[24] Wang Z, Xie L, Ding G, Song S, Chen L, Li G, et al. Single-cell RNA sequencing of peripheral blood mononuclear cells from acute Kawasaki disease patients. Nat Commun. 2021;12(1):5444. 10.1038/s41467-021-25771-5. Epub 2021/09/16. PubMed PMID: 34521850; PubMed Central PMCID: PMCPMC8440575. PMC8440575 34521850

[25] Zhu A, Wei P, Man M, Liu X, Ji T, Chen J, et al. Antigenic characterization of SARS-CoV-2 Omicron subvariants XBB.1.5, BQ.1, BQ.1.1, BF.7 and BA.2.75.2. Signal Transduct Target Ther. 2023;8(1):125. 10.1038/s41392-023-01391-x. Epub 2023/03/17. PubMed PMID: 36922506; PubMed Central PMCID: PMCPMC10015517. PMC10015517 36922506

[26] Jin Y, Jia Z, Cai Q, Sun Y, Liu Z. Escherichia coli infection activates the production of IFN-α and IFN-β via the JAK1/STAT1/2 signaling pathway in lung cells. Amino Acids. 2021;53(10):1609–22. 10.1007/s00726-021-03077-6. Epub 2021/09/16. PubMed PMID: 34524541; PubMed Central PMCID: PMCPMC8441250. PMC8441250 34524541

[27] Niehues P, Wegner FK, Wolfes J, Willy K, Ellermann C, Vollenberg R, et al. Incidence and predictors of cardiac arrhythmias in patients with COVID-19 induced ARDS. J Cardiol. 2022;80(4):298–302. 10.1016/j.jjcc.2022.04.010. Epub 2022/05/20. PubMed PMID: 35589465; PubMed Central PMCID: PMCPMC9108971. PMC9108971 35589465

[28] Chen MY, Xiao FP, Kuai L, Zhou HB, Jia ZQ, Liu M, et al. Outcomes of atrial fibrillation in patients with COVID-19 pneumonia: A systematic review and meta-analysis. Am J Emerg Med. 2021;50:661–9. 10.1016/j.ajem.2021.09.050. Epub 2021/12/10. PubMed PMID: 34879483; PubMed Central PMCID: PMCPMC8483996. PMC8483996 34879483

[29] Kerolos MM, Ruge M, Gill A, Planek MI, Volgman AS, Du-Fay-De-Lavallaz JM, et al. Clinical outcomes of COVID-19 infection in patients with pre-existing cardiovascular disease. Am Heart J Plus. 2022;20:100189. 10.1016/j.ahjo.2022.100189. Epub 2022/08/11. PubMed PMID: 35946042; PubMed Central PMCID: PMCPMC9354393. PMC9354393 35946042

[30] Zuin M, Rigatelli G, Bilato C, Zanon F, Zuliani G, Roncon L. Pre-existing atrial fibrillation is associated with increased mortality in COVID-19 Patients. J Interv Card Electrophysiol. 2021;62(2):231–8. 10.1007/s10840-021-00992-2. Epub 2021/04/16. PubMed PMID: 33855639; PubMed Central PMCID: PMCPMC8046494. PMC8046494 33855639

[31] Nam Y, Jhee JH, Cho J, Lee JH, Shin H. Disease gene identification based on generic and disease-specific genome networks. Bioinformatics. 2019;35(11):1923–30. 10.1093/bioinformatics/bty882. Epub 2018/10/20. PubMed PMID: 30335143. 30335143

[32] Hadjadj J, Yatim N, Barnabei L, Corneau A, Boussier J, Smith N, et al. Impaired type I interferon activity and inflammatory responses in severe COVID-19 patients. Science. 2020;369(6504):718–24. 10.1126/science.abc6027. Epub 2020/07/15. PubMed PMID: 32661059; PubMed Central PMCID: PMCPMC7402632. PMC7402632 32661059

[33] Zhou Y, Frey TK, Yang JJ. Viral calciomics: interplays between Ca2 + and virus. Cell Calcium. 2009;46(1):1–17. 10.1016/j.ceca.2009.05.005. Epub 2009/06/19. PubMed PMID: 19535138; PubMed Central PMCID: PMCPMC3449087. PMC3449087 19535138

[34] Cappellini F, Brivio R, Casati M, Cavallero A, Contro E, Brambilla P. Low levels of total and ionized calcium in blood of COVID-19 patients. Clin Chem Lab Med. 2020;58(9):e171–3. 10.1515/cclm-2020-0611. Epub 2020/05/28. PubMed PMID: 32459190. 32459190

[35] Liao J, Zhang S, Yang S, Lu Y, Lu K, Wu Y, et al. Interleukin-6-mediated-Ca(2+) handling abnormalities contributes to atrial fibrillation in sterile pericarditis rats. Front Immunol. 2021;12:758157. 10.3389/fimmu.2021.758157. Epub 2022/01/04. PubMed PMID: 34975847; PubMed Central PMCID: PMCPMC8716408. PMC8716408 34975847

[36] Bell M, Ekbom A, Linder M. COVID-19 and comedications in atrial fibrillation-a case-control study in Stockholm. Eur J Epidemiol. 2023;38(3):301–11. 10.1007/s10654-023-00967-9. Epub 2023/01/28. PubMed PMID: 36707492; PubMed Central PMCID: PMCPMC9883132. PMC9883132 36707492

[37] Terlecki M, Wojciechowska W, Klocek M, Drożdż T, Kocowska-Trytko M, Lis P, et al. Prevalence and clinical implications of atrial fibrillation in patients hospitalized due to COVID-19: Data from a registry in Poland. Front Cardiovasc Med. 2023;10:1133373. 10.3389/fcvm.2023.1133373. Epub 2023/03/31. PubMed PMID: 36993999; PubMed Central PMCID: PMCPMC10041565. PMC10041565 36993999

[38] Landolfo M, Maino A, Di Salvo E, Fiorini G, Peterlana D, Borghi C. Renin-angiotensin system modulation and outcomes in patients hospitalized for interstitial SARS-CoV2 pneumonia: a cohort study. Intern Emerg Med. 2022;17(5):1335–41. 10.1007/s11739-022-02929-7. Epub 2022/01/23. PubMed PMID: 35064437; PubMed Central PMCID: PMCPMC8782218. PMC8782218 35064437

[39] Zheng PF, Zhou SY, Zhong CQ, Zheng ZF, Liu ZY, Pan HW, et al. Identification of m6A regulator-mediated RNA methylation modification patterns and key immune-related genes involved in atrial fibrillation. Aging (Albany NY). 2023;15(5):1371–93. 10.18632/aging.204537. Epub 2023/03/03. PubMed PMID: 36863715; PubMed Central PMCID: PMCPMC10042702. PMC10042702 36863715

[40] Shi W, Li X, Su Y, Liu D, Wu L, Li S, et al. PILRA is associated with immune cells infiltration in atrial fibrillation based on bioinformatics and experiment validation. Front Cardiovasc Med. 2023;10:1082015. 10.3389/fcvm.2023.1082015. Epub 2023/07/03. PubMed PMID: 37396579; PubMed Central PMCID: PMCPMC10311564. PMC10311564 37396579

[41] Byrd JC, Hillmen P, Ghia P, Kater AP, Chanan-Khan A, Furman RR, et al. Acalabrutinib versus ibrutinib in previously treated chronic lymphocytic leukemia: results of the first randomized phase III trial. J Clin Oncol. 2021;39(31):3441–52. 10.1200/jco.21.01210. Epub 2021/07/27. PubMed PMID: 34310172; PubMed Central PMCID: PMCPMC8547923. PMC8547923 34310172

[42] Graf SA, Lynch RC, Ujjani CS, Gooley TA, Rasmussen H, Coffey DG, et al. Efficacy, safety, and molecular response predictors of oral ixazomib and short-course rituximab in untreated iNHL. Blood Adv. 2023;7(5):687–96. 10.1182/bloodadvances.2022008628. Epub 2022/11/18. PubMed PMID: 36385536; PubMed Central PMCID: PMCPMC9984960. PMC9984960 36385536

[43] Liu X, Peng K, Zhong G, Wu M, Wang L. Bioinformatics analysis of competing endogenous RNA network and immune infiltration in atrial fibrillation. Genet Res (Camb). 2022;2022:1415140. 10.1155/2022/1415140. Epub 2022/08/04. PubMed PMID: 35919038; PubMed Central PMCID: PMCPMC9308555. PMC9308555 35919038

[44] Liu X, Zhong G, Li W, Zeng Y, Wu M. The construction and comprehensive analysis of a ceRNA immunoregulatory network and tissue-infiltrating immune cells in atrial fibrillation. Int J Gen Med. 2021;14:9051–66. 10.2147/ijgm.S338797. Epub 2021/12/09. PubMed PMID: 34876841; PubMed Central PMCID: PMCPMC8643171. PMC8643171 34876841

[45] Zou F, Chen T, Xiang X, Peng C, Huang S, Ma S. Mining of potential biomarkers and pathway in valvular atrial fibrillation (VAF) via systematic screening of gene coexpression network. Comput Math Methods Med. 2022;2022:3645402. 10.1155/2022/3645402. Epub 2022/10/14. PubMed PMID: 36226239; PubMed Central PMCID: PMCPMC9550484. PMC9550484 36226239

[46] Wu R, Fang J, Liu M, Jun A, Liu J, Chen W, et al. SUMOylation of the transcription factor ZFHX3 at Lys-2806 requires SAE1, UBC9, and PIAS2 and enhances its stability and function in cell proliferation. J Biol Chem. 2020;295(19):6741–53. 10.1074/jbc.RA119.012338. Epub 2020/04/07. PubMed PMID: 32249212; PubMed Central PMCID: PMCPMC7212658. PMC7212658 32249212

[47] Bucciol G, Moens L, Ogishi M, Rinchai D, Matuozzo D, Momenilandi M, et al. Human inherited complete STAT2 deficiency underlies inflammatory viral diseases. J Clin Invest. 2023;133(12):e168321. 10.1172/jci168321. Epub 2023/03/29. PubMed PMID: 36976641; PubMed Central PMCID: PMCPMC10266780. PMC10266780 36976641

[48] López-Nevado M, Sevilla J, Almendro-Vázquez P, Gil-Etayo FJ, Garcinuño S, Serrano-Hernández A, et al. Inborn Error of STAT2-dependent IFN-I immunity in a patient presented with hemophagocytic lymphohistiocytosis and multisystem inflammatory syndrome in children. J Clin Immunol. 2023;43(6):1278–88. 10.1007/s10875-023-01488-6. Epub 2023/04/19. PubMed PMID: 37074537; PubMed Central PMCID: PMCPMC10113994. PMC10113994 37074537

[49] MacCann R, Leon AAG, Gonzalez G, Carr MJ, Feeney ER, Yousif O, et al. Dysregulated early transcriptional signatures linked to mast cell and interferon responses are implicated in COVID-19 severity. Front Immunol. 2023;14:1166574. 10.3389/fimmu.2023.1166574. Epub 2023/06/01. PubMed PMID: 37261339; PubMed Central PMCID: PMCPMC10229044. PMC10229044 37261339

[50] Bu LL, Wang HQ, Pan Y, Chen L, Wu H, Wu X, et al. Gelatinase-sensitive nanoparticles loaded with photosensitizer and STAT3 inhibitor for cancer photothermal therapy and immunotherapy. J Nanobiotechnol. 2021;19(1):379. 10.1186/s12951-021-01125-7. Epub 2021/11/23. PubMed PMID: 34802438; PubMed Central PMCID: PMCPMC8607679. PMC8607679 34802438

[51] Jan MW, Chiu CY, Chen JJ, Chang TH, Tsai KJ. Human platelet lysate induces antiviral responses against Parechovirus A3. Viruses. 2022;14(7):1499. 10.3390/v14071499. Epub 2022/07/28. PubMed PMID: 35891479; PubMed Central PMCID: PMCPMC9316291. PMC9316291 35891479

[52] Plens-Galaska M, Szelag M, Collado A, Marques P, Vallejo S, Ramos-González M, et al. Genome-wide inhibition of pro-atherogenic gene expression by multi-STAT targeting compounds as a novel treatment strategy of CVDs. Front Immunol. 2018;9:2141. 10.3389/fimmu.2018.02141. Epub 2018/10/05. PubMed PMID: 30283459; PubMed Central PMCID: PMCPMC6156247. PMC6156247 30283459

