# Supplementary material for: Integrative bioinformatics analysis reveals STAT2 as a novel biomarker of inflammation-related cardiac dysfunction in atrial fibrillation
Source: Open Med (Wars). 2023 Nov 9;18(1):20230834. doi: 10.1515/med-2023-0834 (PMC10655688; doi:10.1515/med-2023-0834)
Supplement: Supplementary material [file med-2023-0834-sm.pdf]

Supplementary material

S1 Materials and methods

S1.2 Data collection and preprocessing from the GEO database

Data were downloaded from the GEO database. The selected dataset details are shown in Table S1. The extracted data were normalized by log2 transformation. The microarray data were normalized by the normalize quantiles function of the preprocessCore package in R software (version 3.4.1). Probes were converted to gene symbols according to the annotation information of the normalized data in the platform. Probes matching multiple genes were removed out from these datasets, The average expression value of gene measured by multiple probes was calculated as the final

Table S1: Datasets used

Dataset	Disease	Case sample	Control sample	Usage here
GSE79768	Atrial fibrillation	14	12	Screening AF DEGs
GSE29819	Atrial fibrillation	0	12	Screening AF DEGs
GSE14975	Atrial fibrillation	5	0	Screening AF DEGs
GSE41177	Atrial fibrillation	32	6	Screening AF DEGs
GSE177477	COVID-19	29	18	Screening COVID-19 DEGs
GSE168732	Kawasaki disease	3	3	Single-cell sequencing analysis
		Sample details		
GSE79768	Case sample (12)	GSM2102184, GSM2102185, GSM2102186, GSM2102187, GSM2102188, GSM2102189, GSM2102190, GSM2102191, GSM2102192, GSM2102193, GSM2102194, GSM2102195, GSM2102196, GSM2102197		
	Control sample (14)	GSM2102198, GSM2102199, GSM2102200, GSM2102201, GSM2102202, GSM2102203, GSM2102204, GSM2102205, GSM2102206, GSM2102207, GSM2102208, GSM2102209,		
GSE29819	Control sample (12)	GSM739016, GSM739017, GSM739018, GSM739019, GSM739020, GSM739021, GSM739022, GSM739023, GSM739024, GSM739025, GSM739026, GSM739027		
GSE14975	Case sample (5)	GSM373956, GSM373957, GSM373958, GSM373959, GSM373960		
GSE41177	Case sample (32)	GSM1005424, GSM1005425, GSM1005426, GSM1005427, GSM1005428, GSM1005429, GSM1005430, GSM1005431, GSM1005432, GSM1005433, GSM1005434, GSM1005435, GSM1005436, GSM1005437, GSM1005438, GSM1005439, GSM1005440, GSM1005441, GSM1005442, GSM1005443, GSM1005444, GSM1005445, GSM1006245, GSM1006246, GSM1006247, GSM1006248, GSM1006249, GSM1006250, GSM1006251, GSM1006252, GSM1006253, GSM1006254		
	Control sample (6)	GSM1005418, GSM1005419, GSM1005420, GSM1005421, GSM1005422, GSM1005423		
GSE177477	Case sample (29)	GSM5374839, GSM5374840, GSM5374841, GSM5374842, GSM5374843, GSM5374844, GSM5374845, GSM5374846, GSM5374847, GSM5374848, GSM5374849, GSM5374850, GSM5374851, GSM5374852, GSM5374853, GSM5374854, GSM5374855, GSM5374856, GSM5374857, GSM5374858, GSM5374859, GSM5374860, GSM5374861, GSM5374862, GSM5374863, GSM5374864, GSM5374865, GSM5374866, GSM5374867		
	Control sample (18)	GSM5374868, GSM5374869, GSM5374870, GSM5374871, GSM5374872, GSM5374873, GSM5374874, GSM5374875, GSM5374876, GSM5374877, GSM5374878, GSM5374879, GSM5374880, GSM5374881, GSM5374882, GSM5374883, GSM5374884, GSM5374885		
GSE168732	Case sample (3)	GSM5160417, GSM5160420, GSM5160422		
	Control sample (3)	GSM5160432, GSM5160434, GSM5160435		

Table S2: Characteristics of the corresponding antibodies included in the context

Antibodies	Origin	SOURCE	IDENTIFIER	Dilutions
HCST	Rabbit	abcam	ab32367	IB 1:1000
STAT2	Rabbit	abcam	ab32367	IB 1:1000
STAT2 (phospho Y690)	Rabbit	abcam	ab191601	IB 1:1000
IRF9	Rabbit	abcam	ab271043	IB 1:1000
RyR2	Rabbit	abcam	ab302716	IB 1:1000
CaMKII (phospho T286)	Rabbit	abcam	ab171095	IB 1:1000
GAPDH	Rabbit	Servicebio	GB11002	IB 1:1000
siRNA	Sequence (5′–3′)			
si*STAT2*[1]	Sense	CCACAUAGCUGAUCUGAAAUU		
	Anti-sense	UUUCAGAUCAGCUAUGUGGUU		

expression value. As in the case of the same dataset and platform but in different batches, used the remove BatchEffect function of the limma package in the R software to remove batch effects. As in different datasets or in the same dataset but in different platforms, extracting multiple data sets with common gene symbols, and marking different datasets or different platforms as different batches, used the remove BatchEffect function of the limma package in the R software to remove batch effects. The PCA plot was drawn to illustrate the samples before and after batch effect. There are 14 AF tissue samples and 12 control tissue samples in the GSE79768 dataset, 12 control tissue samples in the GSE29819 dataset, 5 AF tissue samples in the GSE14975 dataset, 32 AF tissue samples and 6 control tissue samples in the GSE41177 dataset. Moreover, there are 29 COVID-19 PBMC samples and 18 healthy PBMC samples in the GSE177477 dataset. In addition, there are 3 KD PBMC samples and 3 control PBMC samples in the GSE168732 dataset. In our study, the datasets from the GSE79768, GSE29819, GSE14975, and GSE41177 datasets were used to screen DEGs in AF samples by building co-expression networks. The dataset GSE177477 was used to screen COVID-19 DEGs. The dataset GSE168732 was used to validate the function of hub genes in Kawasaki disease. The data procurement and application conform to the GEO databases' principles and guidelines.

S2.2 DEGs identification and functional enrichment analysis

We used the limma package in the R software to study the differentially expressed mRNA. The adjusted *P*-value was analyzed to correct the false positive results in GEO datasets. To further confirm the underlying function of potential targets, the data were analyzed by functional enrichment. Gene Ontology (GO) is a widely-used tool for annotating genes with functions, especially molecular function (MF), biological pathways (BP), and cellular components (CC). Kyoto Encyclopedia of Genes and Genomes (KEGG) Enrichment Analysis is a practical resource for studying gene functions and associated high-level genome functional information. o

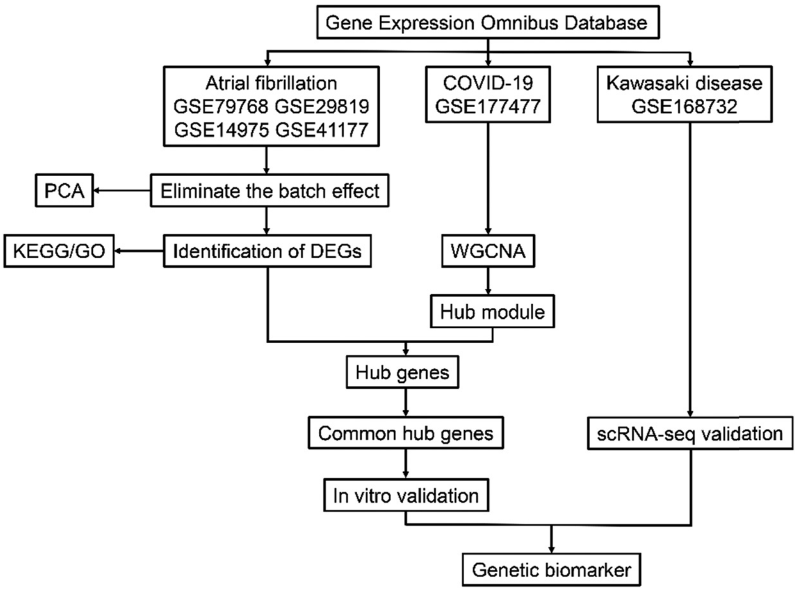

Figure S1: Diagram outlining the step-by-step process of DEG identification and subsequent functional analysis.

better understand the carcinogenesis of mRNA, ClusterProfiler package (version: 3.18.0) in R was employed to analyze the GO function of potential targets and enrich the KEGG pathway; the R software pheatmap package was used to draw heatmap. The DEGs between AF and normal samples, as well as COVID-19 and control samples, were identified using the R packages limma and edgeR1,2. Genes with an adjusted $P < 0.05$ and $|\log2(FC)| > 1$ were selected as DEGs in heart tissues and PBMC samples, respectively. We acquired the shared DEGs using Venny.2, version 2.1.0, an online tool for VENN analysis. Gene Ontology (GO) and Kyoto Encyclopedia of Genes and Genomics (KEGG) analyses were conducted with Clusterprofiler and org.Hs.eg.db3. Terms with a $P < 0.05$ were considered statistically significant. By analyzing DEGs and WGCNA hub genes, we identified COVID-19 and AF shared pathways via Metascape (http://metascape.org/).

S3.2 SARS-CoV-2-Spike-pseudovirus entry assay

All pseudovirus used in this study was purchased from Genomeditech, China (SARS-COV-2 Spike XBB.1.5 pseudovirus GFP-Luciferase). The detailed method was described previously. Briefly, the pseudovirus neutralization was performed as followed: luciferase-expressing pseudovirus was pre-incubated with serially diluted immune sera in 96-well plates for 1 h at 37 °C, following adding the mixture to 293 T/ACE2 cells and then incubating for 48 h to express the reporter gene. The efficiency of viral entry was determined with a firefly luciferase assay. In brief, remove the supernatants of infected cells, then add 50 µl of PBS, 50 µl of lysis reagent from a luciferase kit and luciferase substrate (Promega, USA). Relative light units were performed using a multi-mode microplate reader (PerkinElmer, USA). To treat cardic cell lines, transfected cells were inoculated with pseudovirus for 48h before cells were washed and lysed. In brief, 1×10^6 cells were inoculated into 6cm dishes for 24h, followed by 1µl diluted pseudovirus treatment. In vitro experiments involving SARS-CoV-2 XBB1.5 pseudotyped Virus was performed in Biosafety Level 3 laboratory and strictly followed the approved standard operation procedures. The raw data of pseudovirus was attached to the supplement materials (Table S2 and Figure S1).

Reference

[1] Jin Y, Jia Z, Cai Q, Sun Y, Liu Z. Escherichia coli infection activates the production of IFN-α and IFN-β via the JAK1/STAT1/2 signaling pathway in lung cells. Amino Acids. 2021;53:1609–22.
